# The bergen 4-day treatment for panic disorder: a longer-term follow-up

**DOI:** 10.1186/s12888-025-06527-7

**Published:** 2025-02-07

**Authors:** Thorstein Olsen Eide, Bjarne Hansen, Kay Morten Hjelle, Stian Solem, Michael G. Wheaton, Thröstur Björgvinsson, Gerd Kvale, Kristen Hagen

**Affiliations:** 1 Molde Hospital, Møre og Romsdal Hospital Trust, Molde, Norway; 2 Center for Crisis Psychology, Faculty of Psychology, University of Bergen, Bergen, Norway; 3 Bergen Center for Brain Plasticity, Haukeland University Hospital, Bergen, Norway; 4 Department of Psychology, Norwegian University of Science and Technology, Trondheim, Norway; 5 Department of Psychology, Barnard College, New York, NY 10027 USA; 6 McLean Hospital, Harvard Medical School, Belmont, MA USA; 7 Department of Clinical Psychology, University of Bergen, Bergen, Norway; 8 Department of Mental Health, Norwegian University of Science and Technology, Trondheim, Norway

**Keywords:** Panic disorder, Intensive treatment, Exposure, B4DT, CBT

## Abstract

**Introduction:**

Bergen 4-Day treatment (B4DT) is a form of concentrated exposure-based cognitive behavioral therapy (CBT) in which patients receive treatment over four consecutive days. Previous studies have shown B4DT to be a promising treatment format for panic disorder (PD), although the long-term stability of treatment gains requires additional study.

**Aim:**

The aim of the current study was to evaluate the longer-term effectiveness of B4DT for patients with panic disorder with or without agoraphobia. This study extends a previously published study by providing a long-term follow-up of the same cohort (*n* = 30), initially assessed at three months post-treatment.

**Method:**

Thirty patients with panic disorder were consecutively included in a retrospect open trial. The primary outcome measure was the Panic Disorder Severity Scale. The secondary outcome measures were the Generalized Anxiety Disorder-7 and the Patient Health Questionnaire-9. Outcomes were assessed at pretreatment, posttreatment, 3-month follow-up, and longer-term follow-up (with a mean time of 18 months).

**Results:**

There was a significant reduction in panic disorder symptoms from pretreatment to longer-term follow-up (d = 5.03, 95% CI [18.55, 21.12] to [1.33, 3.87]). The Panic Disorder Severity Scale (PDSS) mean decreased from 19.83 (SD = 0.64, 95% CI [18.55, 21.12]) before treatment, to 4.37 (SD = 0.64, 95% CI [2.98, 5.76]) post-treatment, followed by further decreases at the 3-month follow-up to a mean of 2.84 (SD = 0.64, 95% CI [1.45, 4.22]), and at the longer-term follow-up to 2.60 (SD = 0.64, 95% CI [1.33, 3.87]). There was no significant difference in symptom severity between the 3-month and 18-month assessments, indicating a sustained improvement (*p* <.001). At the 18-month follow-up, 90% of the patients were classified as being in remission. There were also significant reductions in symptoms of depression (*d =* 1.44) and generalized anxiety (*d =* 1.62) that were maintained at the longer-term follow-up assessment.

**Conclusion:**

The results from the current study indicated that the treatment effects of B4DT are stable over time and that the treatment format appears to be promising for PD. Confirming these preliminary results in rigorous study designs is needed.

**Trial registration:**

The study was approved by the Regional Committee for Medical and Health Research Ethics of Northern Norway (REK Nord-2021/209619).

## Introduction

Panic disorder (PD) is an anxiety disorder characterized by recurring unexpected panic attacks, followed by significant maladaptive changes in behavior, such as avoidance of situations and activities that might trigger new panic attacks. Panic attacks are characterized by rapid onset; by intense anxiety; and often by physiological symptoms, such as shaking, sweating, hyperventilating, and racing heart rate [1]. It is estimated that the lifetime prevalence for PD is 3.7% [2]. Compared to unaffected individuals, patients with PD are found to have a significantly lower quality of life, greater occupational difficulties and more frequent use of healthcare services [3]. When untreated PD can be considered a chronic disorder [4]; previous studies have demonstrated little change in wait-list conditions [5, 6], suggesting low spontaneous remission.

Cognitive behavioral therapy (CBT), in addition to pharmacological treatment by selective serotonin reuptake inhibitors, is considered the treatment of choice for patients with PD with or without agoraphobia [7, 8]. Variants of CBT for PD have been demonstrated to be effective in a range of treatment formats [9]. A meta-analysis found no significant differences between group and individual CBT for panic disorder [10]. There is a lack of studies examining the long-term effectiveness of ordinary CBT for PD patients [11, 12]. In addition, the meta-analysis of van Dis et al. [12] found that CBT for panic disorder showed no significant association with better outcomes at 12 + months follow-up (k = 5) One of the longest follow-up studies on PD by Bilet et al. [11] examined patients 24 years after treatment. In this study, 93% reported large benefits from the treatment, and they found a 50% reduction in symptoms on the Phobic Avoidance Rating Scale [11]. Another study by Gloster et al. found a response rate of approximately two-thirds of patients at the 2-year follow-up, with some variation as to what measure was used to classify response [13]. In another study by Addis et al. [14] using the Jacobson and Truax criteria, 36.8% of patients maintained their clinically significant change at the 2-year follow-up.

Several brief, intensive, or concentrated (BIC) treatment formats for CBT have been developed that could make treatment more accessible and possibly more cost effective. These formats usually entail a reduced number of sessions or reduced time between sessions compared to traditional CBT [15]. Previous research on intensified CBT has documented similar treatment outcomes as ordinary CBT [16, 17], and intensified CBT has compared to regular CBT been associated with lower drop-out rates [15], faster treatment response [17] and greater reduction in comorbid symptoms of depression [16]. Importantly, research on intensified CBT has not been thoroughly studied [16].

One form of concentrated CBT that has been developed is the Bergen 4-Day Treatment (B4DT). Originally developed for the treatment of obsessive-compulsive disorder (OCD) [18], The B4DT has demonstrated promising treatment effects in a randomized controlled trial (RCT) for patients with OCD [19]. The B4DT has been adapted to the treatment of PD and showed promising results in a pilot study [20] and when implemented at new treatment sites [21, 22]. Bohni et al. [23] is one of the few studies that has examined the long-term effectiveness of concentrated CBT for PD patients. In this study, the authors found large effect sizes and no significant difference between regular CBT and concentrated CBT at the 18-month follow-up. To date, however, no studies have yet examined the longer-term effectiveness of B4DT, particularly when implementing the treatment at new treatment sites.

The aim of the current study was therefore to examine the longer-term effectiveness of B4DT for PD patients when implementing the treatment format to another clinic than the one in which the intervention was originally developed. We hypothesized that B4DT would show strong treatment effects that would be maintained until the longer-term follow-up assessment. The primary outcome measure was panic disorder severity, which was assessed both in terms of continuous symptom severity and remission status (based on the consensus definition for remission described below). Secondary outcomes included measures of depression and general anxiety.

## Method

### Participants and procedure

The current study was a retrospective naturalistic longer-term follow-up assessment of patients receiving B4DT for PD between October 2017 and January 2020 at the outpatient clinic in Molde, which is a part of the specialist health care in Helse More and Romsdal Hospital Trust in Norway. Building upon a previously published study that reported initial assessments up to a 3-month follow-up [21], this study extends the follow-up duration to 18 months. It included all 30 patients from the original cohort, ensuring a complete retention rate (100%) of the initial sample size.

A summary of demographic and diagnostic information can be seen in Table 1. All patients were assessed and diagnosed pretreatment by an experienced clinician using the Mini International Neuropsychiatric interview (M.I.N.I [24]). Patients over 18 years of age who met diagnostic criteria for PD with or without agoraphobia were included in the study. Exclusion criteria were patients who did not speak Norwegian, were actively suicidal, were experiencing an unstable phase of bipolar disorder, had ongoing psychosis, or were suffering from ongoing substance abuse. 76.7% (*n* = 23) of the patients had both PD and agoraphobia. The majority of the patients (66.7%, *n* = 20) had at least one comorbid disorder and the most common comorbid disorder where recurring with/and current major depressive disorder (MDD) (57.7%, *n* = 17). At the start of treatment 60% (*n* = 18) of the patients used some form of psychotropic medication. The most used medication were SSRIs (36.7%, *n* = 11) and benzodiazepines (26.7%, *n* = 8).The B4DT was administered as part of routine clinical care within Norway’s national public healthcare system. Patients were referred to the outpatient clinic either by their general practitioner or by other qualified mental health clinicians The patients were consecutively referred to the treatment. The study was based on data from a consent-based treatment quality register that obtained ethical approval (REK Nord-2021/209619). Patients were invited to a longer-term follow-up session, where difficulties encountered after the treatment could be addressed. The patients also answered self-report measures either online or on paper during the follow-up session. During the longer-term follow-up session, the Panic Disorder Severity Scale (PDSS) interview was conducted. The PDSS interview was conducted at an average of 18 months (range 12–28 months) after the end of treatment.

**Table 1:** Demographic characteristics

	M(SD)/*N*(%)
Age	38 (13.10)
Previous psychotherapy (Self-reported)	22 (73.3%)
Education level (University/ College)	9 (30%)
Occupational status (Working/studying)	17 (56.7%)
Marital status (Married/in a relationship)	19 (63.3%)
Self-reported duration of disorder (years)	13.43 (14.60)
Gender (proportion female)	21(70.0%)
Self-reported use of psychotropic medication	18 (60.0%)
Benzodiazepines	8 (26.7%)
SSRIs	11 (36.7%)
Comorbidity (any disorder)	20 (66.7%)
Major Depressive Disorder (current or recurrent episode)	17 (56.7%)
GAD	5 (16.7%)
Other anxiety disorders	5 (16.7%)

Note. SSRI = selective serotonin reuptake inhibitor; GAD = generalized anxiety disorder

### Assessment

The PDSS was the primary outcome measure for panic severity in the current study. The PDSS [25] is a seven-item interview rated on a four-point Likert-type scale developed to assess the severity of PD. The interview has shown good psychometric properties and has been demonstrated to be sensitive to change [25]. In the meta-analysis by Papola et al. [26], the PDSS was ranked as the most common measure for assessing symptom severity in PD. We used the criteria developed by Furukawa and colleagues [27] to define clinically significant change and remission. According to these criteria, patients are classified as responders if they have a PDSS score reduction of 40% and as being in remission if they have a reduction of 40% and a score of five or lower, for patients with PD only, or seven or lower, for patients with both PD and agoraphobia. The pre-treatment interview was conducted by the local therapist, while the post-treatment, 3-month, and long-term follow-up assessments were conducted by independent raters.

Secondary outcome measures included self-report measures for depression and symptoms of generalized anxiety. The PHQ-9 [28] is a nine-item questionnaire rated on a four-point Likert-type scale, with scores ranging from 0 to 27 and higher scores indicating higher severity. The PHQ-9 is a widely used self-report measure of depression and has demonstrated good reliability and validity [29]. The GAD-7 [30] is a questionnaire for reporting symptoms of generalized anxiety. The measure has seven items rated on a four-point Likert-type scale, and the total score ranges from 0 to 21, with a higher score corresponding to higher symptom severity. The measure has demonstrated good validity and reliability [30]. The Norwegian versions of the PHQ-9 and the GAD-7 have demonstrated good psychometric properties [31].

### Treatment

The treatment is an intention-focused exposure-based treatment, delivered in this study through the B4DT format. The B4DT is an concentrated treatment format delivered over four consecutive days to groups of 3–6 patients with an equal number of therapists. The treatment format integrates individualized therapist assisted exposure within a group setting [32]. This approach allows for tailored therapist exposure while utilizing the supportive dynamics of group therapy. The treatment format includes standardized psychoeducation in a group setting, complemented by brief therapist meetings throughout the day. This structure is designed to enhance adherence to the treatment protocol and monitor the progress of the individualized treatment plans [33, 34].

Intention-Focused Cognitive Behavioral Therapy (IF-CBT), originally developed by Hansen (second author), posits that discomfort in anxiety disorders largely results from the decisions patients make after perceiving a situation as potentially dangerous, referred to as the patient’s “intention” or “project” [35]. According to IF-CBT, the intention to prevent or avoid feared events leads to emotional activation and discomfort as part of the body’s mobilization to achieve the desired goal. This discomfort is considered less a result of automatic responses or threat-related assumptions and more a consequence of subsequent choices. These choices reinforce the assumption of danger, amplifying future reactions by behaving as if the situation is indeed threatening [35].

This framework is a key component of psychoeducation within IF-CBT. It is illustrated using the Leaning-In Technique (LET), a demonstration that shows how individuals often engage in exposure while still attempting to minimize the risk of feared events or discomfort. LET demonstrates how simple exercises can shift the intention from prevention to letting go or actively causing a feared event [20]. The goal is to conduct exposure in a manner incompatible with the patient’s initial intention or project. Questions such as “Are you still acting as though this is dangerous?” are explored to facilitate this shift. An affirmative answer prompts reflection on whether it is reasonable to expect the body to alter its response patterns under such conditions [32].

Rather than adhering to a predetermined plan for prolonged exposure or behavioral experiments, IF-CBT involves tasks continuously chosen by the patient based on what might help in recognizing and practicing changes to their project or intention. The training often consists of numerous brief tasks, emphasizing that a small task performed correctly is more effective than a longer task without focus on recognizing and altering the project [35].

Day one of the treatment (3–4 h) was dedicated to thorough psychoeducation in a group setting covering PD, maintenance factors and the content, structure and principles of the treatment. During the first day, each patient was assisted in making an individual plan for exposure tasks. Days two and three (7 h each) were dedicated to individually tailored therapist assisted exposure. There were structured group meetings at three points during these days where the patients can share experiences with the group. The time between meetings was dedicated to in vivo exposure to external stimuli that the patients tended to avoid and interoceptive exposure to bodily symptoms that elicit anxiety in the patients. The exposure tasks usually included exercise and hyperventilation, which is intended to increase physical arousal, and exposure in vivo to agoraphobic situations that patients previously avoided. The primary focus in all the exposure tasks was to break previous patterns of avoidance and attempts to regulate anxiety and instead shift to actively approaching and attempting to evoke anxiety. At the end of the second and third day, the patient planned further exposure for the evening and later reported to their therapist over text message. The patients’ relatives or close friends were offered a psychoeducational meeting at the end of the third day. The fourth day (3–4 h) focused on teaching patients how to continue training and maintain treatment effects. The patients were encouraged to make the changes an integral part of their daily lives. The patients thought about important aspects of relapse prevention, and the therapist and patients made an exposure plan for the next three weeks. A more detailed description of the treatment and the therapist can be found elsewhere [21].

### Statistical analysis

The current study had a low amount of missing data. On the longer-term PDSS, there were zero missing interviews. Five patients had missing data for the PHQ-9, and four had missing GAD-7 scores. The expectation-maximization (EM) method in SPSS version 27 was used to implement missing data on the self-report scales PHQ-9 and GAD-7. EM was considered an adept method for imputing missing data when the data were missing at random, and less than 25% data were missing [36]. Little’s MCAR test indicated that the missing data were missing completely at random, *x*^2^ [21] = 18.961, *p =*.588. Repeated measures ANOVA was used to investigate changes from pre- to post, 3-month follow-up, and longer-term follow-up (18 months). When Mauchly’s test of sphericity results were significant, Greenhouse‒Geisser correction was used. For post hoc analysis, Bonferroni correction was used.

## Results

### Changes in symptoms

There were no dropouts from the treatment. Patients showed a significant reduction in PD symptoms over time, as indicated by a repeated measures ANOVA, F(2.269, 65.792) = 230.536, *p* <.001, partial µ^2 = 0.888, with a large effect size, (d = 5.03). Before treatment, the PDSS mean was 19.83 (SD = 0.64, 95% CI [18.55, 21.12]). There was a significant reduction to 4.37 (SD = 0.64, 95% CI [2.98, 5.76]) post-treatment, followed by further decreases at the 3-month follow-up to a mean of 2.84 (SD = 0.64, 95% CI [1.45, 4.22]), and at the longer-term follow-up to a mean of 2.60 (SD = 0.64, 95% CI [1.33, 3.87]), demonstrating sustained improvement (*p* <.001, d = 5.03). No significant changes were observed in PD symptoms from the 3-month to the longer-term follow-up assessments (*p* = 1.00)..

Symptoms of generalized anxiety showed a significant reduction over time. Initially, the mean GAD-7 score was 12.70 (SD = 4.63, 95% CI [10.52, 13.94]). After treatment, the mean score significantly decreased to 5.72 (SD = 3.77, 95% CI [4.18, 6.36]). This reduction continued at the 3-month follow-up with a mean score of 4.96 (SD = 4.77, 95% CI [3.08, 5.89]), and at the longer-term follow-up, the mean score was 5.73 (SD = 4.27, 95% CI [4.00, 7.46]). Statistical analysis confirmed these reductions were significant over time, F(2.454, 71.161) = 483.176, *p* <.001, partial η^2 = 0.511. The large effect size (d = 1.62) indicates a substantial decrease in anxiety symptoms from pretreatment to longer-term follow-up. Importantly, there was no significant change in GAD symptoms between the 3-month and longer-term follow-up assessments (*p* = 1.00), suggesting sustained improvement.

For depressive symptoms, measured by the PHQ-9 scale, there was a significant reduction over time. Initially, the mean PHQ-9 score was 14.40 (SD = 6.50, 95% CI [11.33, 16.11]). After treatment, the mean score significantly decreased to 6.62 (SD = 5.82, 95% CI [4.27, 8.27]). At the 3-month follow-up, the mean score was 6.68 (SD = 6.48, 95% CI [3.98, 8.34]), and at the longer-term follow-up, it was 5.52 (SD = 4.75, 95% CI [3.56, 7.48]). These reductions were significant, F(1.863, 54.037) = 752.824, *p* <.001, partial η^2 = 0.528, with a large effect size (d = 1.44). There was no significant change in depressive symptoms between the 3-month and longer-term follow-up assessments (*p* = 1.00), indicating sustained improvement.

**Table 2:** Results (*M* and *SD*) for the primary and secondary outcome measures (*N* = 30)

Variable	Pre	Post	3 m follow-up	Longer-term follow-up	d = Pre-Longer-term
PDSS	19.83 (3.44)	4.37 (3.72)	2.62 (3.57)	2.60 (3.41)	5.03
GAD-7	12.70 (4.63)	5.72 (3.77)	4.96 (4.77)	5.73 (4.27)	1.62
PHQ-9	14.40 (6.50)	6.62 (5.82)	6.68 (6.48)	5.52 (4.75	1.44

Note. PDSS, Panic Disorder Severity Scale; GAD-7, Generalized Anxiety Disorder-7; PHQ-9, Patient Health Questionnaire-9. d = Cohen’s *d = (M*_*pre*_*– M*_*post*_*)/SD*_*pooled*_

### Treatment response and remission

Table 3 shows the rates of response and remission at longer-term follow-up based on the Furukawa [27] criteria. As shown at longer-term follow-up, 90% (*n* = 27) of the patients were classified as being in remission. Based on the criteria for the PDSS at longer-term follow-up, 86.7% (*n* = 26) of the patients were classified as “very much improved”, while 13.3% (*n* = 4) had “much improved”.

**Table 3:** Status at follow-up was based on criteria from Furukawa et al. [27]

	Posttreatment, *N* (%)	Follow-up, *N* (%)	Longer-term, *N* (%)
Remission	24 (80.0%)	26 (86.7%)	27 (90.0%)
Very much improved (75–100%)	17 (56.7%)	25 (83.3%)	26 (86.7%)
Much improved (40–74%)	12 (40.0%)	3 (10.0%)	4 (13.3%)
Minimally improved (10–39%)	1 (3.3%)	1 (3.3%)	0
No improvement (0–10%)	0	0	0
Missing data	0	1 (3.3%)	0

To examine the change in remission status from the 3-month follow-up to the longer-term follow-up, we examined the individual trajectories of all patients. All patients except three (10%) had no change in their status, and two patients who were not classified as being in remission at the 3-month follow-up had a continued reduction in symptoms and were classified being in the remission category at the longer-term follow-up assessment. One patient (3.3%) who was classified as being in remission at the 3-month follow-up had an increase in PD symptoms and was no longer classified as being in remission at the longer-term follow-up. Of the 30 patients who completed the longer-term follow-up session, two patients requested and received a booster session after the longer-term follow-up session.

## Discussion

This was the first study examining the longer-term effectiveness of B4DT for PD patients. The short-term effectiveness of B4DT for PD patients has been demonstrated in several studies, and the format appears to yield large clinical changes in patients with PD, with low drop-out rates and high treatment satisfaction [20–22]. The current study indicated that the treatment effects are maintained over time. There was a significant reduction in symptoms of PD from pretreatment to longer-term follow-up, and the results from the 3-month follow-up assessment were statistically indistinguishable from those at longer-term follow-up, indicating that the treatment gains were maintained. The large effect size observed for PD symptoms could be inflated as disorder specific measures and uncontrolled study designs are associated with higher effect sizes. However, the study included secondary self-reported outcome measures of depression and generalized anxiety which also showed large effect sizes, although considerably lower than the primary outcome measure. This could indicate that successful treatment of PD is associated with improvement in comorbid conditions and general functioning The large reduction in primary and secondary outcome measure is in line with previous studies on B4DT for OCD patients [32, 37–39], indicating that the treatment format also has a promising effect on longer-term outcomes.

Although the previous findings of research on the B4DT have been in line with other promising research in intensified CBT treatment options, such as the study by Pittig et al. [17], few of the studies on intensified CBT have conducted long-term follow-up evaluations, making comparisons difficult. One of the few studies of intensified CBT that conducted longer-term follow-up assessment is Bohni et al. [23], in which an 18-month follow-up assessment was performed. The authors used the same outcome measure, the PDSS, as the current study and found large treatment effects. As their paper did not report the M and SD for PDSS scores at different assessment points, benchmarking was not possible. However, a comparison between Cohen’s d showed a large difference from *d =* 1.35 to d = 5.03 in the current study. Part of the difference might be attributed to the fact that the current study had higher pretreatment scores, and comparisons with other studies should be made very cautiously, as differences in methods can make comparisons difficult.

There is a lack of studies examining the long-term effectiveness of CBT for PD patients in general [11, 12]. The findings from the current study are in line with previous longer-term findings in the treatment of PD using CBT. A study by Addis et al. [14] examined the longer-term effectiveness of standard CBT and TAU for PD using the PDSS. Compared to the patients in the current study, those study participants had significantly higher PDSS scores at the 1-year follow-up (*p* <.001, *d =* 1.38). This comparison between studies should, as noted earlier, be interpreted with caution, as it is only based on one study, and differences in study design might impact effect sizes.

Another difficulty in comparison and benchmarking findings is that many studies lack uniform outcome measurement and clear criteria for determining response and remission [12]. For instance, one study might use the Phobic Avoidance Rating Scale and an arbitrary definition of 50% reduction as response [11], and another study used the Panic and Agoraphobia Scale (PAS) and a score of 8 or less as a definition of remission [40]. The current study used the PDSS [25], the most common measure for symptom severity in PD [26]. The study also used the criteria for response and remission developed and validated by Furukawa et al. [27], which allows the study to be benchmarked to later research.

The current study had several limitations. Firstly, it was an open trial without control conditions, meaning there was no comparison group to assess the treatment’s effectiveness. The treatment was administered as part of routine clinical care, leading to a lack of adherence monitoring or blinding of the raters. Additionally, the study lacked data on whether patients received other treatments, change of medication, or other important life changes during the follow-up period. The current study lacks self-report measures for PD which is a limitation, However the PDSS have been demonstrated to have a strong correlation with its self-report version the PDSS-SR [41], and to have moderate correlation with other self-report measures for PD [41, 42]. The current study utilized the Norwegian version of the PDSS, whose psychometric properties have not yet been examined. The assessment procedures using the PDSS interview could contributed to the very large effect sizes observed, considering potential sources of bias such as allegiance effects or lack of blinding. Another limitation in the current study is the lack of measurements for function and quality of life. However, the study’s high participation rate of 100% in the longer-term PDSS interview was a notable strength. A notable strength of this study is its implementation in a ‘real-world’ outpatient clinical setting. This gives indications of the potential for adapting the B4DT into regular mental health clinics, it ensures low probability for selection bias and allows for comparisons between studies. Including criteria that matched those used in clinical practice enhances the study’s ecological validity, strengthening the generalizability of the findings to real-world clinical settings. Furthermore, this study was the first to evaluate the longer-term treatment effects of B4DT for PD, yielding promising results. Nonetheless, it is crucial to replicate these findings, and as there have not been conducted an RCT on the B4DT for PD larger, more controlled studies, including randomized controlled trials (RCTs) and adjusted analysis of outcome, are needed to replicate the findings and improve the generalizability of the results.

## Conclusion

The study demonstrated that the effects of B4DT for panic disorder, initially observed at the 3-month follow-up study [21], persisted at the 18-month follow-up. These results suggest potential longer-term stability and underscore B4DT as a promising approach for PD. Further research, including replication studies and larger, more controlled study designs, is warranted to confirm these preliminary findings.

## Data Availability

The dataset for the study is available from the first author upon reasonable request.

## Abbreviations

B4DT: Bergen 4-Day Treatment
CBT: Cognitive Behaviour Therapy
GAD: Generalized Anxiety Disorder
PD: Panic Disorder
PDSS: Panic Disorder Severity Scale
PHQ-9: Patient Health Questionnaire-9
GAD-7: Generalized Anxiety Disorder-7 scale
SSRIs: Selective Serotonin Reuptake Inhibitors
LET: Leaning in Technique
MCAR: Missing Completely at Random
EM: Expectation Maximization
ANOVAs: Analysis of Variance
DSM: Diagnostic and Statistical Manual of Mental Disorders
RCT: Randomized Controlled Trials
TAU: Treatment as Usual
